# Exploring the Long-Term Impact of Emotional Exhaustion on Frontline Nurse Managers Post-COVID-19: A Qualitative Study

**DOI:** 10.1155/jonm/9280686

**Published:** 2025-05-20

**Authors:** David Sanabria-Delgado, Sergio Barrientos-Trigo, Ana María Porcel-Gálvez

**Affiliations:** ^1^Faculty of Nursing, Physiotherapy and Podiatry, Universidad de Sevilla, Seville, Spain; ^2^Virgen Macarena Hospital, Seville, Spain; ^3^Research Group PAIDI-CTS 1141 “Clinical Research Applied to Care and New Care Paradigms (ICCAPA)”, Andalusian Public Health Service, Seville, Spain; ^4^Research Group PAIDI-CTS 1050 “Complex Care, Chronicity and Health Outcomes”, Andalusian Public Health Service, Seville, Spain

**Keywords:** burnout, coronavirus infections, hospitals, nurse managers, psychological

## Abstract

**Aim:** To explore emotional exhaustion in frontline nurse managers after 3 years of COVID-19, considering their essential role in healthcare systems and the prolonged impact of the pandemic on staff well-being and organizational effectiveness.

**Design:** A qualitative phenomenological study was used. This approach attempts to uncover the essence of the experiences that nurse managers may have had.

**Method:** Semi-structured interviews were conducted with 12 frontline nurse managers at a tertiary-level university hospital in Spain during May 2023. The data were analyzed using thematic analysis. A thematic framework was developed, and coding was guided by a well-established methodological approach.

**Results:** Five categories were established after the analysis: general difficulties related to fear and uncertainty, and continuous changes in protocols, availability of human resources, accessibility to material resources, management carried out with relatives of patients, and emotional management.

**Results:** Following the analysis, five key categories were identified that reflect the main challenges faced by nurse managers: (1) general difficulties related to fear and uncertainty, aggravated by frequent changes in protocols and the work environment; (2) availability of human resources, marked by high absenteeism and work overload; (3) accessibility to material resources, where the shortage of protective equipment and medical supplies generated ethical dilemmas and operational tensions; (4) management with patients' relatives, a significant emotional component that required balancing empathy and safety measures in high-conflict contexts; and (5) emotional management of managers, which evidenced a significant emotional impact, highlighting the need for clear strategies to prevent burnout and foster resilience in these critical roles.

**Conclusions:** Nursing managers experienced emotional exhaustion during the pandemic, not only due to the health consequences of the virus but also due to the complex management of material, human, and family resources. They faced difficult situations with families justifiably separated from their vulnerable loved ones. This highlights the need for specific interventions, such as psychological support, leadership training, and better allocation of resources, to reduce the risk of burnout and strengthen a more resilient healthcare system.

**Summary**

• Patient or public contribution
◦ The interviewees participated in the interpretation of the data.

## 1. Introduction

COVID-19 caused a worldwide health and social crisis [1]. The crisis was characterized by high healthcare pressure, lack of protective equipment, and increased infections in healthcare professionals, leading to higher rates of absenteeism from work. This situation led to a deterioration in the psycho-emotional health of healthcare professionals [2], who reported high levels of work-related stress and emotional trauma, that affected their motivation and coping strategies [3]. After more than 3 years since the first cases, it has become a long-distance race that exhausted health staff. Currently, studies on pandemic psychiatry show an increase in cases of post-traumatic stress and other health problems in this population [4].

COVID-19 challenged health systems worldwide. According to WHO data, the United States, India, and Brazil recorded the highest number of accumulated infections since the beginning of the pandemic [5]. This contagion increased especially among health workforce, a pillar crucial for health system resilience [6]. More specifically, nurses range between 38.6% and 48% [7].

Among frontline nurses, nurse managers played a critical role in hospital management during the pandemic [8]. Studies exploring the vision of frontline nurse managers look at managing nurse redeployment before and during the COVID-19 pandemic and how to improve management of ongoing nurse redeployment [9]. Other similar studies look at appropriate human resource management to increase nurse productivity and quality of care [10].

Nurse managers combine leadership and management in an integrated manner, playing a key role in team coordination, planning, and continuous improvement of care. Their responsibilities include setting strategic directions, aligning and motivating staff, and managing resources through organization, planning, and problem-solving. They are instrumental in creating a positive work environment, fostering interprofessional collaboration, and supporting the personal and professional development of nurses. Additionally, they implement evidence-based practices, structure daily work, and manage staff recruitment and support, all aimed at optimizing nursing care outcomes [11].

Being a nurse manager requires a lot of effort and dedication. All complaints and claims from the staff reach them. However, these complaints serve to realize what is happening and thus have the opportunity to improve [12, 13]. The available evidence highlights personal characteristics, competence, education, and social support as factors that influence the behaviors of frontline nurse managers in leadership capacity. Mentoring and peer support programs or work environments that support relational and structural support could increase the leadership capacity of these nurses [14].

Nurse managers encountered significant barriers, including fear of contagion, excessive workload, and ethical challenges related to managing scarce resources [15]. Their vulnerability to emotional exhaustion (EE) is heightened by the dual responsibility of overseeing both material and human resources in a high-pressure environment characterized by uncertainty and high demand. As mediators between staff and administration, they must make critical decisions under pressure, redeploy personnel amid mass absences, and resolve ethical dilemmas while maintaining team morale and managing the emotional impact of restrictions on patients' families [16, 17]. These challenges align with those faced by nurse managers during health crises or disasters, where the need to balance leadership, emotional support, and operational efficiency increases the risk of burnout. Expanding strategies that promote resilience is essential to safeguarding their well-being and ensuring their effectiveness in critical situations [18]. A high prevalence of depression, anxiety, insomnia, and post-traumatic stress disorder among healthcare workers has been identified [19]. All this also caused an accelerated professional exhaustion process among frontline nurse managers after 3 years of pandemic [20].

EE, together with depersonalization (DP) and low personal accomplishment (PA), is one of the three constructs that form the basis of burnout syndrome. The WHO stresses that burnout does not necessarily require impairment of all three dimensions and emphasizes EE as the main characteristic [21]. Leiter and Maslach, in their analysis of organizational predictors, also argue that impairment of a key dimension, such as EE, may be sufficient to justify preventive burnout interventions [22].

To date, studies on the pandemic have addressed the EE of frontline healthcare professionals [23–25] or the initial experiences of nurse managers [26, 27], but none have analyzed the long-term impact of challenges such as resource rationing, absenteeism, or patient–family dynamics on nurse managers. There is a gap in the literature, as no study on the long-term impact on nurse managers was found. This longitudinal study on EE in nurse managers is significant because this period allows for a more in-depth analysis of the long-term effects of the health crisis on their emotional and professional well-being. This temporal approach provides a more complete perspective on how initial and ongoing experiences have influenced their levels of EE. Furthermore, studying this phenomenon in a later context allows for the identification of key learnings, changes in work dynamics, and emerging needs, offering a solid foundation for developing sustainable strategies for institutional support and prevention of EE in the future. Therefore, the aim of this study is to explore EE in frontline nurse managers after 3 years of COVID-19 through their experiences, perceptions, and feelings.

## 2. The Study

The aim of this study was to explore EE in frontline nurse managers 3 years after the onset of COVID-19. In general, nurses suffered from different symptomatologies associated with posttraumatic syndrome after the COVID-19 pandemic. These symptoms ranged from insomnia or anxiety to feelings of guilt or sadness. In the case of nurse managers, these feelings are more related to EE. Therefore, it would be interesting to explore how these feelings are expressed.

## 3. Methods

### 3.1. Design

A qualitative phenomenological study was conducted using an empirical approach and a hermeneutical–dialectical method [28]. This methodology allows exploring the experiences, perceptions, and feelings that can be deeply rooted among nursing professionals [29] and thus also determining whether the pandemic has favored the appearance of EE among the population studied.

Phenomenology allows us to explore experiences as they are lived and perceived by the participants in our study, understanding the meaning they have in the context of those who live them [30]. With the hermeneutic–dialectical method, we can also analyze complex phenomena that involve multiple perspectives, such as the relationship between professionals, patients, and organizational structures [31, 32]. Both approaches allow us to understand and address complex phenomena related to health care, work dynamics, and the experiences of professionals. Phenomenology and the hermeneutic–dialectical method can effectively complement each other in a qualitative study of nursing by providing a comprehensive framework for exploring and understanding participants' lived experiences in a specific context, such as patient care or nursing management. The method helps to obtain meaningful results in qualitative research [33].

The study was conducted at a tertiary-level university hospital in Spain that provides the basic needs of specialized care for almost 500,000 citizens [34]. This hospital belongs to the Andalusian Health Service (SAS). It is attached to the Ministry of Health and performs the functions assigned to it under its supervision and control [35]. It is a hospital with highly specialized technical staff and equipment and highly differentiated clinical services by function [36]. This hospital had the highest rate of COVID-19 infections among its professionals compared to other hospitals in that city [37].

In this study, an implicit triangulation was established based on observation, interview, document analysis, and other forms of data collection that are pragmatically combined [28]. Subsequently, a triangulation was also carried out with the other researchers in the study and with the Maslach Burnout Inventory (MBI).

### 3.2. Sample and Recruitment

A convenience and intentional sampling was performed [28], where nurse managers of the frontline units used for COVID-19 patients were included. The following inclusion criteria were established: (1) nurses who were in this management position for at least 12 months; (2) managers whose units housed COVID-19 patients even if they were intermittent; and (3) managers who continue in the position at the time of the study. Among the exclusion criteria, we included nurse managers who left management during the pandemic or those who started management once the pandemic began. Initially, it was considered to exclude all those nurse managers who might have psychological after-effects associated with their experience, but finally, all the frontline nurse managers were chosen. The main researcher had in-depth and close knowledge of the nurse managers at this hospital, particularly those who worked on the frontline during the COVID-19 pandemic.

Contact was made directly with these nurse managers. No one refused to participate. The study sample consisted of 12 nurse managers (7 women and 5 men), aged between 37 and 63 years ($\overset{¯}{x}$ = 51.4), with whom data saturation was reached. Participants included three nurse managers from COVID hospitalization units, two from the Intensive Care Unit (ICU), two from the emergency department, one from pediatrics, and four bed managers. Only 25% (*n* = 3) had postgraduate management training, with an average of 11.5 years of managerial experience. We ensure the diversity of the sample by selecting nurse managers of different sexes, different years of management experience, and different units, all of them on working frontline during the COVID-19 pandemic.  Table 1 presents the sociodemographic and academic characteristics of the participants.

### 3.3. Data Collection

The information was collected through semi-structured interviews conducted in May 2023. Previously, a bibliographic review was carried out to see the suitability of the research, prepare the interview script, and establish the a priori categories. For the latter, the personal experience of the researchers in this study was also contributed. Following the planned topics, we formulated the established open questions. The interviews were dynamic; we were able to use our creativity based on the different participants, and they provided flexibility to adjust the questions to said participants (Appendix A: Interview Guide). Additionally, the MBI was administered to the interviewees.

A preliminary pilot of the interview script was conducted to adjust the questions and thus ensure that they would meet our objective. The pilot interview revealed strengths and weaknesses in the interview design. We found that the questions generated the expected answers, in-depth and of quality, but we adjusted the order of the questions and the setting, which could not be a work setting. All interviews were conducted individually by the main researcher at the time preferred by the informants. Previously, there was a relationship with the interviewees before the study because they work in the same hospital as the researchers. The environment was ensured to be the most suitable, and the privacy of the interviewees was maintained. The interviews were held outside the workplace. There were no other people present, in addition to participants and researchers. Although field notes were taken during the interview, the audio of the interviews was recorded using a digital recorder to avoid distractions and interruptions in the free flow of communication and to avoid losing information due to the limited memory of the interviewer [38]. The mean duration of the recordings was 40 min (range: 25–55). The transcripts were made between June and July 2023. To increase the validity of the statements, we used peer checking by sending the transcripts back to the interviewees to confirm that they felt they identified with the content.

To avoid excessive fragmentation [39], and considering that the volume of data is manageable [40], software was not used to process the data obtained, but manual analysis was performed for both methodological and practical reasons. Manual analysis allows the researcher to interact directly and deeply with the data [30, 41] and encourages a more reflective and contextualized engagement with it [31], which is relevant in studies where a deep understanding of human experiences, interactions, and subjective meanings is paramount.

### 3.4. Data Analysis

For data analysis, we employed the general qualitative data analysis process proposed by Rodríguez et al. This approach provides a structured method for qualitative analysis, enhancing the reliability of the findings. This framework consists of three phases: (1) data selection and reduction, involving the identification of relevant information from the raw data while eliminating redundant or non-essential elements; (2) data organization and transformation, where selected data are systematically categorized and structured to facilitate interpretation; and (3) obtaining and verifying conclusions, a phase in which emerging patterns, themes, and relationships are identified and validated to ensure analytical rigor. This process is iterative and reflexive, allowing the researcher to adjust and deepen their analysis [42].

**Figure 1:**
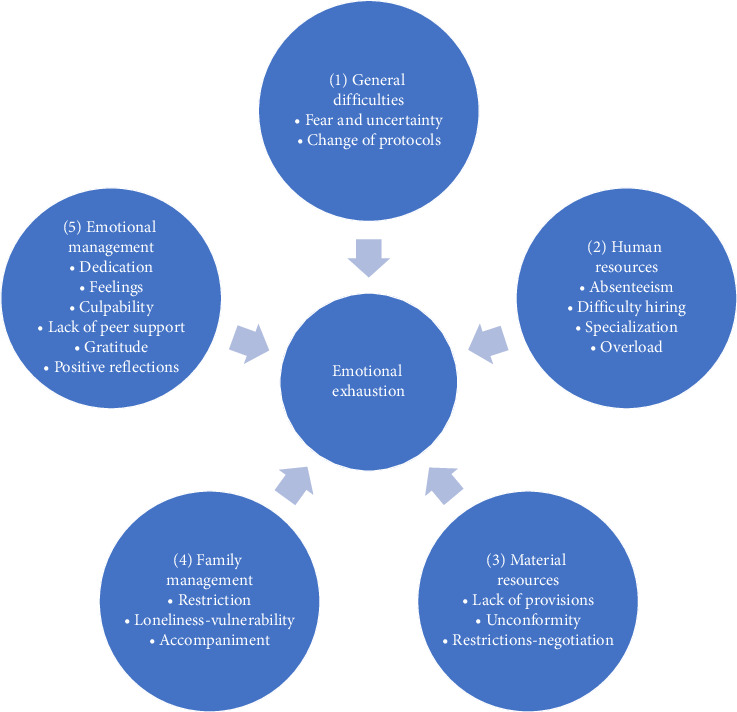
Framework of categories and their effects on burnout syndrome.

Data were analyzed using thematic analysis. An initial framework of thematic ideas was established, including broad categories that were then refined as the analysis progressed [43]. The text was then categorized using concept-guided coding, following Gibbs' proposal, in three phases: initial reading, coding without restricting oneself to predefined categories, and coding into broader categories based on conceptual similarities and connections identified in the text. The use of concept-guided coding ensured a rigorous and informed analysis [44]. This coding was carried out by the interviewer and another researcher. The final categories emerged through an iterative process in which recurring patterns and relationships between categories were analyzed [45]. The established analytical categories are shown in Figure 1.

### 3.5. Rigor

Initially, we considered the Lincoln and Guba's trustworthiness criteria to ensure the rigor of the study. However, we ultimately relied on Calderón's criteria, since this procedure considers the most contemporary methodological advances and debates. Therefore, we chose a coherent epistemological approach and explained the chosen qualitative design (epistemological adequacy), selected a significant problem (relevance), reached data saturation and triangulated it (validity), and reflected on possible personal and contextual biases (reflexivity) [46]. At the same time, we placed greater emphasis on interpretive depth and methodological clarity [47].

To mitigate interviewer bias, a researcher was selected from within the research team who had a close relationship with the interviewees, sharing the same professional category and management position, as well as several years of employment relationship with them. This choice facilitated an environment of trust that promoted the comfort of the interviewees and favored the sincere expression of emotions and feelings. In addition, another member of the research team participated exclusively in the data analysis phase, with the aim of reducing possible biases in the interpretation by the interviewer.

Furthermore, to ensure the quality of the study, we have included the Consolidated Criteria for Reporting Qualitative Research (COREQ) checklist [48]. In addition, the results of the qualitative study were triangulated with the results of the MBI scale applied to the sample. We integrated the MBI scale at the end of the interviews. The results provided complementary information to our study. While the qualitative interviews delved deeper into the subjective and contextual aspects of our object of study, the MBI scale offered a more quantitative and general view.

The MBI consists of 22 items that measure burnout in terms of EE (nine items), DP (five items), and PA (eight items). It is measured on a seven-point Likert´s scale anchored by never (0) and every day (6). The scores thus can range from 0 to 54 on the EE subscale, from 0 to 30 on the DP subscale, and from 0 to 48 on the PA subscale. Higher mean scores on the EE and DP subscales correspond to higher levels of burnout, whereas lower mean scores on the PA subscale correspond to higher levels of burnout [49]. Total scores of each dimension were summed up and categorized as low, moderate, or high. In order to do so, high, moderate, and low scores were considered as ≥ 27, 19–26, and ≤ 18, respectively, in EE, ≥ 10, 6–9, and ≤ 5, respectively, in DP, and ≤ 33, 34–39, and ≥ 40, respectively, in personal performance [50]. The cutoff values indicating the presence of burnout were ≥ 27 for EE, ≥ 10 for DP, and ≤ 33 for PA, which was scored in the opposite direction [51].

**Figure 2:**
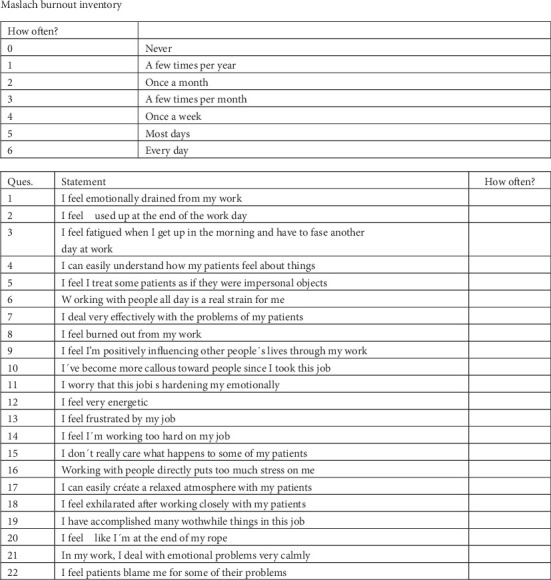
MBI questionnaire distributed to respondents. Copyright Mind Garden, Inc. [52].

This scale includes items such as “I feel emotionally drained from my work at the end of the day,” which assesses EE by measuring the extent to which professionals experience fatigue and depletion due to their workload. For DP, an example item is “I sometimes treat patients as if they were impersonal objects,” reflecting a detached or cynical attitude toward patients, a core characteristic of burnout. Finally, PA is evaluated with items like “I feel I am positively influencing other people's lives through my work,” which captures the professional's sense of efficacy and fulfillment in their role. These items collectively contribute to the comprehensive assessment of burnout in healthcare professionals (Figure 2: MBI scale).

### 3.6. Ethical Considerations

All participants were previously informed about the subject of study. All signed the informed consent, keeping the informative copy. We made sure that the interview did not cause any traumatic memories. Each interview was recorded in audio format to later make a literal description. The audio files were kept by the main researcher and were eliminated once the transcriptions were made, maintaining the anonymity and confidentiality of the data. An alphanumeric code was assigned to identify each of the interviews and to preserve the privacy of the participants. This research was approved by the Research Ethics Committee of the Virgen Macarena-Virgen del Rocío University Hospitals (Code: 0609-MI-21).

## 4. Results

**Figure 3:**
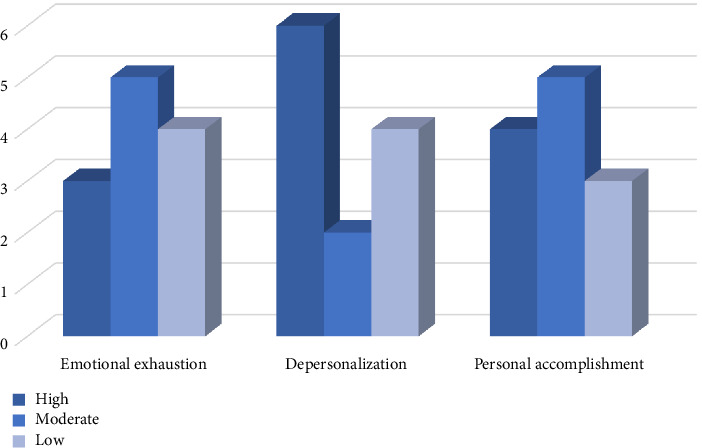
Descriptive of MBI.

The MBI shows that 25% of the interviewed nurse managers exhibited high levels of EE, 50% reported high levels of DP, and 25% demonstrated a low level of PA. The descriptive results of the MBI are illustrated in Table 2 and Figure 3.

Five thematic categories were identified to group nurse managers' speeches: (1) general difficulties related to fear and uncertainty, and continuous changes in protocols, (2) availability of human resources, (3) accessibility to material resources, (4) management carried out with relatives of patients, and (5) emotional management. All interviewees talked about the same topics, without any minor ones. (1) In crises such as the pandemic, nurse managers face fear, uncertainty, and pressure from critical decisions with limited information, aggravated by frequent changes in protocols that generate insecurity and affect team cohesion. (2) Human resource management is a key challenge for nurse managers, as staff shortages, sick leave, and work overload create conflict and affect team morale, especially during crises such as the pandemic. (3) The shortage of material resources, such as protective equipment, generates stress, ethical dilemmas, and tensions in the team, especially in health crises, affecting confidence in management and the quality of care. (4) Managing family members involves balancing empathy and safety measures, a challenge intensified in crises such as the pandemic, where restrictions have complicated communication and generated tensions that fall on managers. (5) Nurse managers experience a variety of feelings, due to the high pressure and continuous responsibility in times of crisis, where their resilience and institutional support are essential to sustain their work.

### 4.1. General Difficulties

During crisis situations, such as the COVID-19 pandemic, nurse managers face an environment marked by fear of exposure to the virus, uncertainty about its evolution, and the constant pressure to make critical decisions with limited information. This is exacerbated by frequent changes in protocols, which require rapid adaptation and create insecurity among staff. Furthermore, fear and uncertainty can impact team cohesion, as professionals face varying levels of stress and anxiety.

#### 4.1.1. Fear and Uncertainty

An element that is repeated in all the interviews and that marked all the professionals interviewed is the existence of fear and uncertainty on the part of all health personnel, for not knowing about this virus and its consequences.

“We have had to deal with situations of fear, ignorance, distrust, anguish and anxiety” (S5).

“The greatest difficulty was in the beginning, the concern and fear of professionals, when everything was unknown and the form of transmission was ignored” (S11).

“It was fear, it was panic, because nobody knew what we were facing…” (S3).

#### 4.1.2. Change of Protocols

A peculiarity of the first wave that was reflected by the interviewees was the continuous change of protocols. This was mainly due to initial ignorance of the pathophysiology of the disease and perhaps also to the limited provision of material resources:

“The difficulty of adapting to changes in COVID protocols, which at the beginning of the pandemic varied from day to day” (S5).

“The greatest difficulty that I have seen has been the confusion of professionals due to the continuous changes in the protocol” (S10).

In conclusion, nurse managers face high levels of fear, uncertainty, and pressure in healthcare crises, which affected both their well-being and team cohesion, highlighting the need for organizational support in these contexts.

### 4.2. Human Resources

Human resource management is one of the main challenges for nurse managers, especially in high-demand settings. Staff shortages can lead to work overload and constant redistribution of tasks, leading to frustration and conflict within the team. Nurse managers must balance efficient staff allocation with the need to maintain team morale and well-being, facing sick leave, frequent turnover, and lack of specialized staff. This issue is even more critical in situations such as a pandemic, where EE and prolonged absences directly affect the quality of care.

#### 4.2.1. Absenteeism

A visible problem that has been established throughout this time of the pandemic is absenteeism, especially after the second wave, due to infections and quarantines due to close contacts. Subsequently, absenteeism caused by secondary effects of vaccines was added to these:

“Those staff who got sick or quit and there was not enough. We have had some difficulty there” (S6).

“Added to this was the difficulty in managing absenteeism of the staff when in close contact with patients in the units or with other professionals…” (S5).

#### 4.2.2. Difficulty Hiring

During the second wave, the difficulty of the lack of nurses for their hiring was added, although only at the beginning:

“They couldn't hire more because there were no more nurses, so have you ever seen yourself…with a rope around your neck?” (S1).

“Sometimes, due to high casualties and the lack of personnel in the stock market to hire, coverage was compromised” (S2).

#### 4.2.3. Specialization

The lack of training of contracted professionals was also observed. This is due to ignorance of the pandemic itself and the need for specialized professionals in the different units where more nurses were needed:

“This shows that the specialty in intensive care is needed…I think it has been shown more than ever in the pandemic” (S3).

“Nursing specialization has been lacking. People with very little experience in pediatrics have come […] Very new people who just finished their degree” (S9).

#### 4.2.4. Overload

At the same time, this entails an extra burden for veteran staff, who has to train new and inexperienced staff, and most importantly, has an impact on health outcomes:

“The lack of knowledge of the staff has been an overload for the old staff, right? They are super burnt out. And that has repercussions on the patient, it has repercussions on the usual professionals of the unit, on the results…” (S3).

“That lack of personnel that we had in a very, very complex service was compensated for by colleagues who signed up for hours and that load was accumulating” (S8).

In conclusion, human resource management was a key challenge for nurse managers, especially in high-demand settings, where staff shortages and workloads affected both team morale and quality of care.

### 4.3. Material Resources

Lack of adequate access to material resources can lead to stress and compromise the quality of care. Nurse managers must make strategic decisions to prioritize the use of scarce resources, which can lead to ethical dilemmas and tensions with staff. This issue becomes especially critical in health crises, where demand outstrips availability. Resource restrictions can also erode staff trust in management and increase feelings of vulnerability.

#### 4.3.1. Lack of Provisions

Material resources are something that was also mentioned by all nurse managers. During the first wave, it was difficult to provide the staff with material. Although, in the end, there was always material, there were many experiences that nurse managers had.

“That uncertainty was what meant that we should have comprehensive control on a daily basis” (S1).

“There were days of uncertainty, at first, not knowing if the material we had would arrive at the end of the week. I was more rationed, but protection was never lacking in my units” (S2).

“The biggest difficulty we have encountered has been the lack of material […] especially at the beginning of the pandemic” (S12).

#### 4.3.2. Unconformity

Although there was material provision, professionals adopted a nonconformist attitude, mainly due to ignorance of its effectiveness and, therefore, lack of confidence in it:

“Everything was counted, but there was no lack, it was unconformity, with everything a total disagreement” (S4).

“The lack of resources has not been the problem, but the dissatisfaction of professionals with the resources they received. Unconformity because they thought that with what there was, one was not well protected” (S3).

#### 4.3.3. Restrictions-Negotiation

The initial restrictions made with material resources were intended to ration stocks in the most effective way and thus to be able to provide all personnel with protective equipment. Although it could sometimes appear to be a negotiation, this served so that there was always a supply:

“There was a time when we had to market with masks […] The centralized warehouse management, quite restrictive, is to be able to have response capacity in the later days due to the lack of supply we had from outside. And here, in that aspect, with all the shortcomings that have been, we have come out quite quite quite well” (S8).

In conclusion, the lack of adequate material resources, especially in health crises, generates stress, ethical dilemmas, and tensions in the team, which affected both the quality of care and trust in management.

### 4.4. Family Management

Interaction with patients' families represents a significant emotional and organizational component for nurse managers. In critical situations, such as during the pandemic, the need to restrict family access complicated communication and created additional tensions. Nurse managers must balance empathy toward family members with the implementation of safety measures, which requires clear and sensitive communication skills. In addition, managing family members' uncertainty and emotions involves dealing with conflictual situations, which often falls to the leadership of nurse managers.

#### 4.4.1. Restriction

Another important issue is that of family management, mainly from two points of view. The first is the management of a family that has been limited in terms of accompanying patients due to the imposed health measures. Security was supported and access was restricted. This has to a certain extent facilitated the work of professionals, who with fewer influxes of relatives were able to apply higher quality care:

“As there are fewer family members, the number of requests for information decreases. This means that the professional can spend more time with the patient and that the care provided is better quality” (S6).

“[…] the security system has also changed, so right now the security guards are going around, many more times than before […]” (S9).

“[…] they have realized that it is better to be COVID unit because there are no relatives, it is more comfortable to work” (S11).

#### 4.4.2. Loneliness-Vulnerability

However, the second point of view of family management, and more important, is dealing with a family that was not able to accompany its relatives in critical moments of health. This was a source of EE for nurse managers, who have empathized with this difficult situation of isolated patients:

“It is the feeling of abandonment that the patient himself has […] I would feel that way, right? Vulnerable, very vulnerable” (S7).

“Many times you turn fragile patients into those who are not […] The fact of being in a room incommunicado with your family who cannot come to see you…is difficult to manage” (S8).

“I have seen that family members were distressed because they could not see their patients […] and that the anxiety they had, the truth…was difficult to treat” (S12).

#### 4.4.3. Accompaniment

However, both in the COVID hospitalization unit and in the ICU, great effort was made to allow family support of highly dependent and/or critically ill patients, always observing security measures.

“We have always given the option to the families of highly dependent patients or those with a critical prognosis to accompany their families” (S2).

“At the beginning of the pandemic, visits were prohibited, nobody could pass, and that was terrible. It was terrible for us, obviously terrible for the family, terrible for the patient, but we immediately revoked, contrary to everything most ICUs still do, we revoked that immediately” (S3).

In conclusion, interaction with patients' relatives is an emotional and organizational challenge for nurse managers, especially in crises, where they must balance empathy and security, managing tensions and conflicting emotions.

### 4.5. Emotional Management

Nurse managers' subjective experiences and perceptions are often marked by a mix of EE and a sense of ongoing responsibility toward their team and patients. These experiences reflect how managers perceive their role as leaders in times of crisis, facing ethical dilemmas, difficult decisions, and high levels of pressure.

#### 4.5.1. Dedication

All participants in this study expressed how their EE has increased during this pandemic period. This was a consequence of the number of hours these nurse managers dedicate to management work. If under normal conditions, nurse managers dedicate more time than their working day; during this time, they made an enormous effort.

“[…] some beastly workloads. There were days when I came home at eleven at night and sat at the computer to finish a protocol” (S3).

“We have lived here from seven in the morning to ten at night every day, from Monday to Sunday, without days off […] We have abandoned our families” (S7).

#### 4.5.2. Feelings

Many are the feelings that nurse managers expressed related to their EE, from impotence, tiredness, worry, or despair:

“The pressure was brutal in terms of the information they demanded of us, the mood was very tense […] they made you responsible for the pressure they were suffering” (S6).

“What has burnt me the most has been the lack of empathy of many colleagues. A great lack of empathy for the office” (S8).

#### 4.5.3. Culpability

Nurses, immersed in this situation of uncertainty, fear, and bewilderment, blamed the nurse managers for all the lack of material, as well as for the infections between professionals. They qualify this as one of the most important factors causing their EE:

“They attacked you, blamed you, blamed the management, blamed the politicians, but you are the one who stood up and the truth is that it was…it was tremendous” (S3).

“The basic professional, then, made you responsible for not being supplied with the appropriate material […] He made you responsible for something that you were not really responsible for, that colleagues were infected…” (S6).

#### 4.5.4. Lack of Peer Support

Many of the testimonies obtained in the interviews refer to the treatment received by their own colleagues and staff nurses. On many occasions, they felt misunderstood, disappointed, and saddened by the attitudes of some colleagues who suffered from the psychological desolation caused by this pandemic, this being one of the most decisive factors in the process of exhaustion suffered by managers.

“COVID has brought out the worst in people […] This lack of resources has brought out the worst in many people […] We are more burnt out by the professionals themselves than by the work itself” (S9).

“Professional fatigue has been brutal. […] The pressure to which we have been subjected has been enormous. […] The staff nurse was subjected to a lot of pressure, almost as if I were talking about mass hysteria, and that pressure was transmitted to you […] We have also suffered insults, pressure, and that is leaving the sequel” (S6).

“It has been the biggest disappointment in the world, because I have felt completely frustrated, because every time we took a step to improve something, there were always voices (staff nurses) who threw it on the ground or blocked you” (S8).

#### 4.5.5. Gratitude

One of the most rewarding things and one that helped most alleviate this exhaustion of nurse managers was the expressions of gratitude received, both from some colleagues and family members.

“I have been congratulated, I have been congratulated by many, many people who come from outside (nurses from other hospitals). […] It's been a bit of a boost, hasn't it?” (S3)

“And yet, after all, they tell you…“it is true, you have helped us”” (S8).

“Even the families of patients who have died have been grateful, they have congratulated the work, the support, the fact that they have been able to live it as something more or less normal, to be able to fire him” (S3).

#### 4.5.6. Positive Reflections

Finally, it is worth highlighting some expressions that shed light on this pandemic in the midst of so much darkness. Positive reflections and optimistic attitudes emerged in the relentless struggle. The best face is shown at the worst possible time because you learn from everything.

“We have made more strengths and fewer weaknesses […] And despite that, we have promoted messages on social networks that praise our work and how good we are” (S8).

“I feel tired, but not frustrated, because these hard months have been a life lesson. Facing the COVID monster has meant getting closer to the suffering of the professional, the patient, and the family and, above all, realizing that I am surrounded by magnificent nursing professionals who have given everything, putting patient care before their fears” (S5).

“This year has not wreaked havoc on me. Yes, we have had a bad time. There have been specific situations where you have collapsed. But I think I have known how to recover and continue fighting” (S12).

In conclusion, nurse managers managed their emotions during the pandemic, facing diverse feelings such as guilt, gratitude, and the need to maintain positive thoughts. This emotional management was key to sustaining their great dedication and resilience, allowing them to lead teams with empathy and effectiveness in a challenging environment.

## 5. Discussion

The objective of this study was to explore the prevalence of EE in nurse managers through their experiences, perceptions, and feelings after 3 years of the COVID-19 pandemic. For this, we used a qualitative methodology that has allowed us to explore the causes of EE of nurse managers.

66.6% of nurse managers reported high-moderate levels of EE. However, only 25% had a high level. Regarding DP, 66.6% also reported high-moderate levels, with 50% having a high level. Although these results reported by the MBI scale are not very high, their lived experiences indicate a notable increase in EE. Their perceptions, feelings, and testimonies express a situation of tiredness, exhaustion, and disappointment. Qualitative interviews allow us to explore lived experiences, underlying meanings, and emotions in a broad and dynamic context [53]. These can reveal nuances, contradictions, or aspects that the scale does not capture. This may also be because the scales measure EE at a specific time, while interviews allow us to explore the emotional and work trajectory of the participants. This can lead to discrepancies if current feelings do not fully reflect accumulated experience [40].

The high levels of DP and EE reported in the MBI are closely related to the challenges nurse managers face in their daily work, especially during the COVID-19 pandemic. The fear and uncertainty inherent in crisis situations, coupled with the constant management of limited human and material resources, significantly increase the stress and emotional fatigue of nursing leaders. Work overload, staff shortages, and tensions with patients' families can exacerbate EE. Furthermore, the subjective experiences of nurse managers, marked by the feeling of continuous responsibility and the constant facing of ethical dilemmas, reinforce vulnerability to EE. Higher levels of EE have been observed in nurse managers working in critical areas such as the ICU, inpatient units, and emergency departments, compared to those in pediatrics. This may be explained by the prevalence of the impact of COVID-19 on the adult population, which represents the majority of hospitalized patients during the pandemic. Additionally, nurses with higher seniority in management roles tend to report higher levels of EE, possibly due to the accumulation of stress inherent to their prolonged responsibilities in high-pressure settings.

Nursing managers experienced higher levels of EE during the COVID-19 pandemic compared to other healthcare professionals due to their specific role. Unlike professionals who focus exclusively on direct patient care, nursing managers have the additional responsibility of mediating between staff needs and management demands. In addition, they faced constant pressure to maintain team morale while dealing with the fear of contagion, uncertainty, and long working hours, which significantly increased their emotional and psychological burden [54].

These factors contribute to the risk of EE, which is exacerbated by the sense of responsibility toward their teams and patients, as well as the need to make quick decisions in a context of uncertainty. Research indicated that this EE is also related to structural barriers and lack of sufficient support during critical phases of the pandemic [54].

The initial difficulties identified by nurse managers at the beginning of the pandemic coincide with fear and uncertainty in other studies, due to the daily risk of exposure to the virus, due to problems of access and use of personal protective equipment, as well as work overload and a higher demand for patient and family care [55]. However, according to our study, the demand from families decreased due to the adopted health restrictions.

One of the characteristics of nurse managers is the profound dedication, the multitude of unpaid hours that they used for the hospital. Many of our participants expressed difficulties reconciling work and family during this time of pandemic. Being a nurse manager requires a high level of personal commitment, reflected in long working hours that exceed working hours and therefore separate you from your family. Lack of motivation makes it difficult to stay in such a demanding job [12]. This generates consequences in nurse managers, such as a high degree of perceived stress associated with high burnout rates that is offset by acceptable job satisfaction [13]. On the other hand, the individual affective commitment of the nurse manager is an important quality to maintain a good relationship between nurses, as well as with other nurse managers or with the physician. It is essential to develop an identification with the work unit [56].

The lack of material resources, or the uncertainty about their supply, caused confrontations between care nurses and their managers. And this was expressed, violently and unassertively, to the nurse managers. Nurses can be very demanding emotionally. These can help caregivers reduce stress and prevent EE that often leads to lack of enthusiasm and motivation at work. Additionally, the nurse manager must also create a relaxed and trusting environment [57].

The demands placed on nurse managers by nursing staff resulted in significant EE. Although it is a complaint to a higher hierarchy, nurse managers act as a barrier between care nurses and their management. They are a link between management and professionals and are responsible for generating the necessary motivation and attitude for greater efficiency in practice [58].

The common element that integrates human resources and health outcomes is the competence of professionals [58]. The knowledge, skills, and abilities of the professional will be closely related to health outcomes. An important issue starts with this, highlighted by some nurse managers who recognized, during the pandemic, the need for specialization in critical care. The complexity and acuteness of the patients in these units require specialized care, creating difficulties for nurses and physicians [59]. Bloomer et al. state that most nurses providing critical care patient care should recognize certification in this specialty [60]. This pandemic highlighted the urgent need to create the specialization of the nursing in intensive or emergency care [61, 62]. This problem not only occurs in Spain, but, together with new priorities, clinical problems, and other global events and influences, it affects critical care nursing, making it a worldwide challenge [63].

Seventy-five percent of the nurse managers interviewed did not receive specific postgraduate management training. González García et al. show how to reach the highest level of the role of nurse manager in Spain; master's and doctorate studies are necessary [64]. Several authors state that the professional role of nurse managers is in the process of transformation and that one of their main demands is more training opportunities [65].

Restrictions on the presence of relatives of hospitalized patients yielded dichotomous results. On the one hand, nurses' managers expressed that the quality of care improved because nurses were able to spend more time with patients and less time with family members. On the other hand, many patients found themselves alone, vulnerable, and fragile, which is why humanizing care work gained vital importance. This had also an emotional impact on professionals, and the good of accompaniment became more important than the risk of contagion to the family member [66].

Finally, in this time, professionals made clinical decisions that, due to their complexity, they do not know if they were correct. This and all the aforementioned difficulties had a significant impact on the mental health of healthcare professionals who cared for these patients and their families [67]. Different studies show the need to create specific mental health programs to minimize emotional impact, offering recommendations such as physical exercise, breathing exercises, or requesting professional help [2]. Other studies highlight the need for psychological care during the pandemic and in subsequent phases incorporating a gender perspective and using evaluation instruments to assess the clinical evolution of health professionals throughout the crisis [68].

### 5.1. Limitations

As a limitation, we found the difficulty in conducting the interviews. Everything experienced by nurse managers, especially at the beginning of the pandemic, they experienced with a great traumatic component, so we had to apply mechanisms to avoid traumatic memories in nurse managers. There was a previous survey necessary based on decisive feedback. The sample was sufficient because it ensured the saturation of the data. However, although this study does not intend to generalize the findings, it would be interesting to carry out this investigation with the frontline nurses at other hospitals.

These findings could serve to identify EE as a key variable in situations of intense stress or even post-traumatic stress in management, such as in crisis management, care for multiple victims, high-frequency management, even other future pandemics. By monitoring this variable, it would be possible to anticipate possible short- and long-term consequences, implementing preventive or rehabilitative interventions that would reduce the impact on managers. Future research could study the effectiveness of interventions that reduce EE in managers after health crises.

### 5.2. Implications for Policy and Practice

This study serves to provide visibility into the work of nurse managers, in which the pandemic has been shown to favor the appearance of EE among the studied population. This research could serve to create new categories that could be included in future quantitative research. At the same time, it can help prevent EE for nurse managers in upcoming pandemics. The findings show keys to improving difficult management with staff and with the family and thus avoid EE. Similarly, the findings show them how nurse managers must have staff with the necessary skills to deal with upcoming pandemics, including specialized staff to improve patient outcomes.

These findings can be applied in practice by promoting the implementation of emotional support programs for nurse managers, as well as ensuring the availability of adequate material and human resources in crisis situations. Furthermore, it would be beneficial to develop clear and flexible protocols that allow for more efficient and less draining management in similar future scenarios. From a forward-looking perspective, the emotional impact of the pandemic underlines the importance of strengthening emotional resilience and leadership skills in nurse managers, as their role will be crucial in managing future crises. Establishing peer-support networks could provide a foundation for shared learning and mutual encouragement during challenging periods. This learning could also inform policies to train managers in anticipating and managing crises, prioritizing both staff well-being and the quality of care provided. Additionally, incorporating leadership training focused on crisis management and developing mental health interventions tailored specifically to the needs of managers would further equip them to navigate complex and emotionally demanding situations.

## 6. Conclusions

EE of nurse managers increased not only due to the idiosyncrasies of the virus but also due to complicated management with material and human resources and with the family. The main difficulties are lack of materials or inaccessibility and professionals immersed in a situation of fear and uncertainty with high absenteeism. In addition, support for families had a strong emotional impact on nurse managers. Interviewees highlight the need for specialized nursing in critical care.

The findings can be applied by promoting emotional support programs and ensuring adequate resources for crisis nurse managers. Furthermore, it is essential to develop clear and flexible protocols to facilitate efficient management. Prospectively, the emotional impact of the pandemic highlights the need to strengthen the resilience and emotional leadership of nurse managers, informing policies that prioritize their training and well-being along with the quality of care.

Longitudinal studies may be needed to track the evolution of EE over time, providing a dynamic perspective on the factors that contribute to its onset and persistence. Cross-cultural comparisons would also be valuable to explore how nurse managers' experiences may vary in different sociocultural and organizational contexts, providing a more comprehensive understanding of the cultural influences on the perception and management of EE.

## Figures and Tables

**Table 1:** Sociodemographic characteristics of the participants.

Code	Gender	Age	M.S. and F.S.	Unit	M.E.	C.R.	P.T.
S1	Female	41	Married with children	COVID hospitalization	1	Eventual	No
S2	Female	37	Single without children	COVID hospitalization	1	Interim	No
S3	Female	54	Married with children	ICU	15	Permanent	Expert
S4	Female	63	Divorced with children	ICU	30	Permanent	No
S5	Female	52	Single without children	Bed manager	11	Permanent	No
S6	Male	49	Divorced without children	Bed manager	4	Interim	No
S7	Female	50	Divorced with children	Emergency	11	Permanent	Grades
S8	Male	57	Married with children	Emergency	1	Permanent	No
S9	Male	56	Married without children	Pediatrics	17	Permanent	Grades
S10	Male	59	Married with children	Bed manager	30	Permanent	No
S11	Female	41	Married with children	COVID hospitalization	2	Interim	No
S12	Male	58	Married with children	Bed manager	15	Interim	No

Abbreviations: C.R. = contractual relationship, F.S. = family situation, M.E. = management experience in years, M.S. = marital status, and P.T. = postgraduate training in management.

**Table 2:** Results of MBI.

MBI	S1	S2	S3	S4	S5	S6	S7	S8	S9	S10	S11	S12
E.E.	26 moderate	6 low	33 high	26 moderate	11 low	50 high	31 high	24 moderate	24 moderate	21 moderate	6 low	9 low
D.P.	8 moderate	3 low	12 high	2 low	1 low	27 high	13 high	18 high	6 moderate	10 high	3 low	12 high
P.A.	34 moderate	38 moderate	30 low	41 high	37 moderate	28 low	37 moderate	33 low	39 moderate	42 high	44 high	41 high

## Data Availability

The data that support the findings of this study are available from the corresponding author upon reasonable request.

## Appendix A: Interview Guide

1. What have been the main challenges identified in healthcare management during the last 3 years of the pandemic, and how have they impacted the efficiency of the system?
2. Based on your experience, how has the lack of material resources influenced the development of professional activities during the pandemic?
3. Had aspects of human resource management in the context of the pandemic have been effective, and which have generated greater difficulties?
4. How would you describe the experience of managing interaction and communication with families of patients?
5. What factors have contributed to the increase in emotional fatigue in healthcare personnel during the last 3 years of the pandemic?
6. In relation to your professional experience, could you describe the predominant emotions in this period, including possible emotions of exhaustion, frustration or burnout, and their possible causes?
